# Case report: Phosphoinositide 3-kinase inhibitor with fulvestrant in a patient with ER+/HER2- metastatic breast carcinoma induced fatal arrhythmias: a preventable event?

**DOI:** 10.3389/fonc.2024.1331472

**Published:** 2024-06-17

**Authors:** Li Zhang, Yanlei Zheng, Gao Chen, Fang Zhao, Shi Li

**Affiliations:** ^1^ Department of Intensive Care Medicine, Hubei Cancer Hospital, Tongji Medical College, Huazhong University of Science and Technology, Wuhan, China; ^2^ Department of Cardiology, ZhongNan Hospital of Wuhan University, Wuhan, China

**Keywords:** phosphoinositide 3-kinase inhibitor, QT/QTc, atrioventricular block, torsade de pointes, electrocardiogram

## Abstract

Phosphoinositide 3-kinase (PI3K) inhibitors have shown synergistic anticancer effects with endocrine therapy against ER+/PIK3CA-mutated breast cancer. PI3K inhibitors for cancer therapy are becoming more common. There is an increasing need to understand their cardiac adverse events. In this report, we describe the features of near-fatal mixed arrhythmias in a patient who was undergoing a phase Ib clinical study of PI3Kα inhibitor with fulvestrant. Subsequently, the patient survived by cardiopulmonary resuscitation and therefore did not die. This case highlights that PI3K inhibitors can induce QT/QTc prolongation and predispose patients to TdP. The combination of QT/QTc prolongation in combination with prolonged cardiac repolarization, such as an AV block during treatment with PI3Kα inhibitor, may aggravate the occurrence of TdP. It is likely to be a safer strategy to adjust the standard of discontinuing drugs and continuing drugs (QTc interval was <500 and <60 ms at baseline) or choose other types of alternative treatment options. This report provided some ideas for clinicians to identify early and prevent the occurrence of fatal arrhythmias during anticancer treatment.

## Introduction

With different kinds of new anticancer drugs emerging, many heart-related adverse drug reactions (ADRs) have appeared (1). One crucial and potentially harmful marker is prolonged cardiac repolarization, which is reflected by a prolonged QT interval in the ECG (1, 2). Prolongation of the QT can develop into severe ventricular arrhythmia of the torsade de pointes (TdP), which can induce sudden death (3). The phosphoinositide 3-kinase (PI3K) pathway plays a key role in cell growth, survival, apoptosis, metabolism, and myocardial contractility (4, 5). The combination therapy of PI3K inhibitors has presented a better clinical activity particularly in patients who have PIK3CA-mutated tumors (6). PI3K signaling could control cardiac repolarization and increase action potential duration by regulating multiple ion channels (7, 8). However, mixed arrhythmias which are caused by PI3K inhibitors have not been reported previously. This report provides clinical empirical evidence, which we hope will help healthcare practitioners to identify and prevent potential severe arrhythmia during treatment with PI3K inhibitors.

## Case presentation

**Figure 1:**
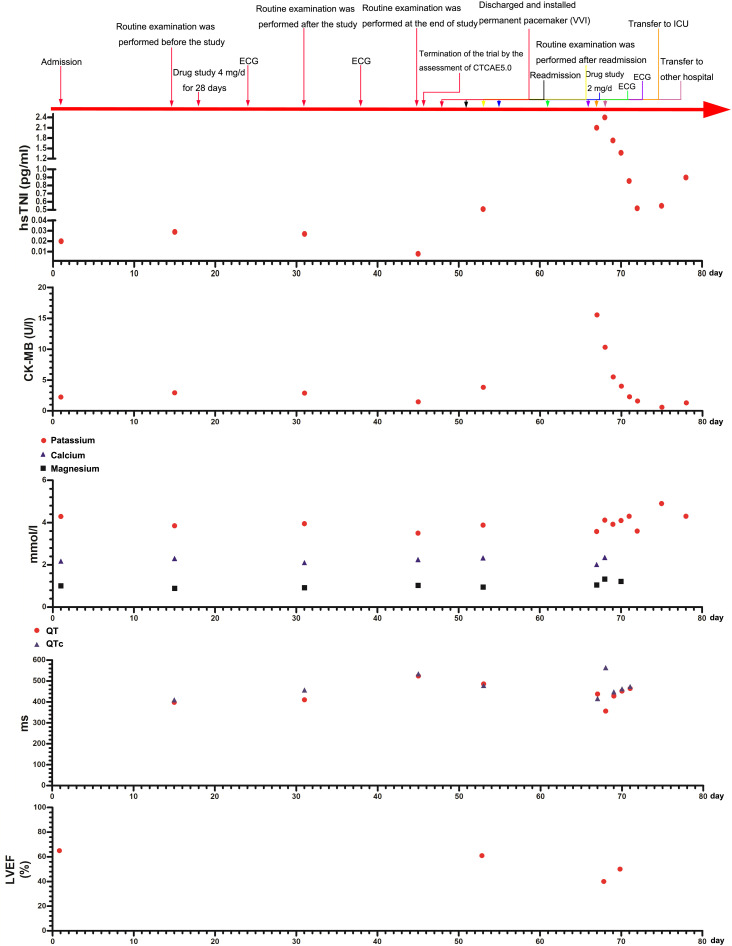
Main clinical and laboratory findings and therapeutic interventions from admission on day 0 to discharge from the ICU on day 78 are depicted. The QT interval correction method is after the Bazett formula. hs-TNI, hypersensitive troponin I; CK-MB, creatine kinase-MB; LVEF, left ventricular ejection fraction.

**Figure 2:**
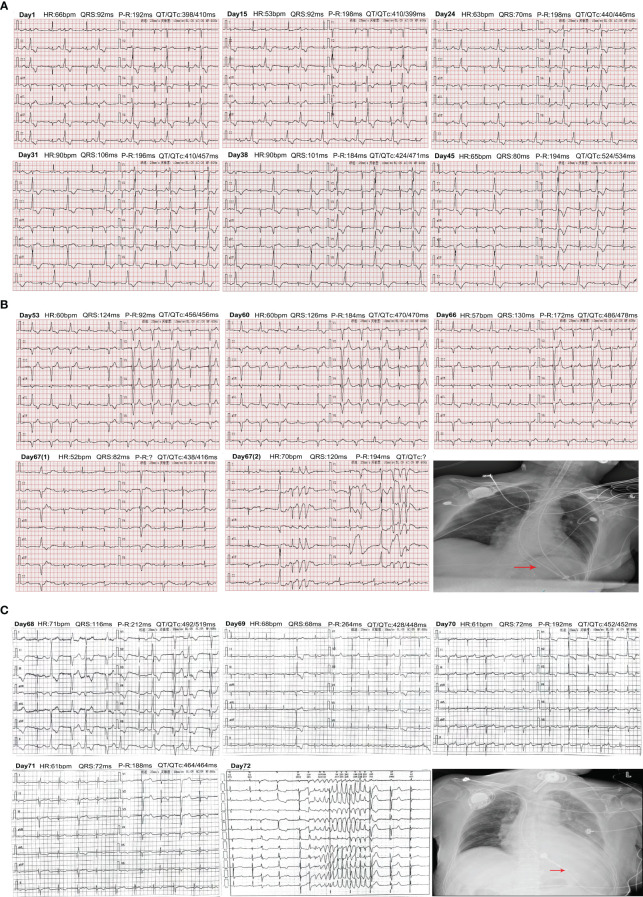
Changes of electrocardiogram from admission to discharge. **(A)** Changes in the ECG from admission to before the patient was installed. Days 1–24: ECG showed QT/QTc interval gradually prolongation. Days 31–45: ECG displayed worsening QT/QTc interval and atrioventricular block. **(B)** Changes in the ECG from enrolling in the study again to transfer to our hospital ICU. Days 53–66: ECG showed gradual prolongation of the QT/QTc and worsening of AV block. Day 67: Changes of ECG after being transferred to the ICU. The chest X-ray revealed that the location of the cardiac pacemaker was normal after CPR. **(C)** Changes in the ECG and chest X-ray in the patient after transfer to another hospital’s ICU. Days 68–71: ECG changes from days 1 to 4. Day 75: The occurrence of TdP was recorded on the 5th day. The chest X-ray displayed that the location of the cardiac pacemaker was normal.

A 73-year-old woman had a radical mastectomy for right breast cancer 6 years earlier and had no other history. This was followed by oral letrozole (2.5 mg/day) for approximately 5 years, oral exemestane (2.5 mg/day) for 8 weeks, oral chidamide (30 mg/3 day) for 4 weeks, and discontinuation of the drug 3 months before admission. The carcinoma recurred after a series of anticancer therapies failed. The patient’s repeated immunohistochemistry test results were as follows: ER (40%, +), PR (-), HER2 (0), KI67 (LI: 40%), and PI3KCA mutation. The initial laboratory tests were in the normal range (**Table 1**; **Figure 1**). A 12-lead ECG showed premature ventricular contraction (PVC), frequent bigeminy, and QT/QTc (398/410 ms) (**Figure 2A**). The patient met the project criteria of a phase Ib study of PI3K inhibitor with fulvestrant in PI3KCA-mutated metastatic breast cancer (project number: HS-10352–102), signed the informed consent, enrolled in the clinical study, and started to take the drug orally (4 mg/day). She took it for 28 days. The patient had no chronic disease, as only the trial drug was taken orally during the study. The ECG revealed frequent PVC, frequent bigeminy, and QT/QTc (440/446 ms) a week after the start of the study (**Figure 2A**). The laboratory results were in the normal ranges, but the ECG revealed QT/QTc of 410/457 ms, frequent PVC, frequent bigeminy, and second-degree AV block 2 weeks after the study (**Figures 1A**, **2A**). The initial AV block was assessed as CTCAE V5.0 grade 1–2, and the study continued. We still found frequent PVC, frequent bigeminy, second-degree AV block, and QT/QTc (424/471 ms) 3 weeks after the study started (**Figure 2A**). At 4 weeks, the ECG showed QT/QTc of 524/534 ms and an almost complete AV block (**Figure 2A**). The second-degree AV block turned to an almost complete AV block, which was assessed as CTCAE V5.0 grade 3. The patient immediately withdrew from the clinical study and had a permanent pacemaker implanted.

**Table 1:** Patient data and laboratory findings on admission.

Years	73	
Gender	Female	
Chief compliant	Right breast cancer surgery 6 years ago, and right breast recurrence 9 months	
Past medical history	Occasional premature atrial contraction and premature ventricular contraction	
Drug therapy	Intravenous AC-T chemotherapy for 8 weeks after surgery Oral letrozole(2.5mg/day) for approximately 5 years Intravenous nab-P drugs(100mg/3weeks) for 6 weeks Followed oral Exemestane(2.5mg/bid) for 56days followed oral chidamide(30mg/3days) for 28days	
Blood pressure	135/71mmHg	
	Normal range	value
Leukocyte count	3.5-9.5×10^9^/L	5.1
Neutrophil count	1.8-6.3×10^9^/L	2.15
Lymphocyte count	1.1-3.2×10^9^/L	2.22
Platelet count	125-350×10^9^/L	200
Erythrocyte count	3.5-5.5×10^12^/L	3.23
Prothrombin time	9.4-12.5s	13.5
Activated partial Thromboplastin time	25.1-36.5s	32.3
D-dimer	0-0.5mg/L	2.82
Creatin kinase	18-198U/L	88
Creatine kinase-MB	0-5ng/mL	2.55
Hypersensitive troponin I	0-0.04ng/ml	0.019
Brain natriuretic peptide	0-100pg/ml	21.66
Alanine aminotransferase	0-40U/L	13.3
Aspartate aminotransferase	0-40U/L	17.1
Total bilirubin	0-20umol/L	8.91
Blood urea nitrogen	2.8-7.6mmol/L	4.7
Blood glucose	3.9-6.1mmol/L	5.39
Creatinine	41-111umol/L	50
Calcium	1.97-2.85mmol/L	2.18
Potassium	3.5-5.4mmol/L	4.29
Sodium	135-148mmol/L	141.5
Magnesium	0.75-1.25mmol/l	1.01
Lung CT scan	Right chest wall tumor and abnormal density shadow of right ribs	

We implanted a single-chamber permanent pacemaker (VVI). The ECG revealed QT/QTc of 456/456 ms, normal pacemaker sensing, and an almost complete AV block (**Figure 2B**). The laboratory results were also in the normal range. CTCAE V5.0 grade 3 turned to grades 1–2, so we adjusted the dose of the PI3K inhibitor (2 mg/day) and kept her on it for 28 days (**Figure 1**). We still observed a gradual prolongation of the QT/QTc (470/470 ms) after a week, QT/QTc (486/478 ms) after 2 weeks, and a consistently complete AV block (**Figure 2B**). The patient suddenly lost consciousness with no pulse after 12 days of dose adjustment. Continuous cardiopulmonary resuscitation (CPR) was performed, and the patient was transferred to the ICU. The patient’s circulation eventually recovered with short episodes of ventricular tachycardia (VT), and TdP frequently occurred (**Figure 2B**). The chest X-ray result revealed that the location of the cardiac pacemaker was normal (**Figure 2B**). We achieved hemodynamic stability with a continuous infusion of magnesium sulfate (4 mg/min) and isoprenaline (1–3 μg/min), and the patient maintained a normal potassium level.

The patient was transferred to the ICU of another hospital for further antiarrhythmic therapy on the next day. The echocardiography showed a weakened systolic function and LVEF of approximately 40% (**Figure 1**). The laboratory tests results are shown in **Figure 1**. The ECG showed prolongation of the QT/QTc, frequent PVC, and almost complete AV block for 4 days (**Figure 2C**). The patient still had occasional short episodes of TdP on the 5th day, but the myocardial enzymes were gradually declining, and the chest X-ray result revealed that the location of the cardiac pacemaker was normal (**Figure 2C**). Finally, the patient recovered consciousness, was weaned from mechanical ventilation, and was discharged from the ICU after 2 weeks.

## Discussion and conclusion

Numerous clinical reports have shown that PI3K inhibitors combined with endocrine therapy in HR+/HER2- advanced breast carcinoma could prolong progression-free survival (PFS) and enhance the overall response rate (ORR), clinical benefit rate (CBR), and median overall survival (OS) compared with control treatments (9, 10). According to FDA drug instructions, monitoring blood glucose and glycosylated hemoglobin before and after treatment with PI3K inhibitors is important (11). One study reported an incidence of hyperglycemia of 65%, an incidence of diarrhea of 58%, and a non-infectious pneumonia onset of 1.8% during alpelisib treatment. However, its adverse cardiovascular drug reactions are not reported in the clinic.

The patient had no chest pain or other cardiac symptoms, but the occurrence of fatal arrhythmias seems to be traceable. She could be observed to have experienced a second-degree AV block approximately 2 weeks after the start of the study, and the second-degree AV block worsened to almost a complete AV block throughout the study. Whether PI3K inhibitors induce AV block is unknown and has rarely been reported. The effect of PI3K inhibitors on AV block is permanent or temporary and requires extensive clinical data. We also observed a gradual prolongation of the QT/QTc during the study. ECG revealed a QT/QTc (524/534 ms) of approximately 120 ms above baseline and complete AV block after 4 weeks. We observed the QT/QTc (456/456 ms) to be below 60 ms from baseline and complete AV block after the pacemaker was implanted. Her cardiac risk seemed to have been removed, only to develop cardiac arrest when she had an adjusted drug dose. We recorded three ECG changes of QT/QTc gradual prolongation, which eventually led to cardiac arrest. There is no threshold of QTc prolongation at which TdP is certain to occur. The QTc greater than 500 ms has been associated with a twofold to threefold higher risk for TdP, and each 10-ms increase contributes to approximately 5% to 7% exponential increase in risk (12). Therefore, it appears that it is difficult to predict when to stop the PI3K therapy. We found (1, 2) no evidence showing that fulvestrant can lead to QT prolongation, so we can speculate that PI3K inhibitors lead to the prolongation of the QT/QTc. Whether it is correlated with the dose of the drug is unknown. The drug effects on cardiac conduction function require further clinical data and pharmacokinetic studies.

We might find some clues to prevent the occurrence of fatal arrhythmia in this case. Firstly, we should fully assess the medical history, including older age (>65 years), sex, a congenital long QT syndrome (LQTS), a high baseline QTc, hypothyroidism, and induced QTc prolonged by other drugs (1, 2). Secondly, the risk classification of anticancer drugs and supportive drugs should also be fully estimated, and evaluation of the risk of QTc prolongation is necessary for moderate–high risk drugs (13, 14). Thirdly, monitoring the ECG and electrolyte levels is also crucial and the easiest way to get it. The latter report (1) also emphasized the standard of discontinuing the drugs when the QTc interval was ≥500 or ≥60 ms above baseline until the QTc interval was <500 or <60 ms above baseline. Electrolyte imbalances directly increase the risk of developing TdP, just as several drugs that cause QTc prolongation corrected the target value for serum K+ from 4 mEq/L to the upper limit of normal, and serum Ca^2+^ and Mg^2+^ within the normal range was safer (15).

We found a special phenomenon in which both the QT/QTc interval and AV block were gradually exacerbated in this case. We also noticed that the patient had frequent bigeminy before the pacemaker was implanted. There are reports that bigeminy can be another arrhythmia contributing to torsades (16). Although we cannot predict whether a patient will experience sudden cardiac death, the mixed arrhythmias should be noticed as early as possible. Some reports (17, 18) have shown that AV block-induced cardiac electrical and structural remodeling predisposes the heart to TdP in an AV dog model. Bhattad et al. (2023) (19) described that tachycardia usually shortens the QTc interval, but the adjustment is not instantaneous. Bradycardia increases the risk of QTc prolongation and therefore the risk for torsade de point. It is therefore logical that a patient with increasing AV block picture and subsequent bradycardia has an increased risk for torsade de points. Bradycardia and torsade de points are connected. The mechanism by which AV block and PI3K inhibitors induce TdP is that both can lead to prolonged cardiac repolarization and further promote action potential prolongation (18). We observed that the patient still experienced multiple short episodes of TdP when the QTc interval was <500 and <60 ms at baseline. Thus, we believe that the previous standard of discontinuing drugs and continuing drugs no longer seems to be safe.

The report stresses a comprehensive assessment of risk factors for QT prolongation before anticancer therapy. Frequent recording of ECG changes and electrolyte levels is necessary during the treatment. When the QT was gradually prolonged along with this type, arrhythmias of cardiac repolarization and action potential prolongation appear together. It should be of great concern to healthcare professionals. It is likely to be safer to choose other types of alternative treatment options or downregulate the standard for discontinuing and restarting drugs. No more data were collected from the other patients participating in the trial. We only report this one case, and there was no serum concentration monitoring in this case. We frequently monitored the patients’ ECG and found three kinds of arrhythmias. It seems that it could provide some proof for clinicians to identify early and prevent the occurrence of fatal arrhythmias during anticancer treatment.

## Data availability statement

The original contributions presented in the study are included in the article/supplementary material. Further inquires can be directed to the corresponding authors.

## Ethics statement

The studies involving humans were approved by the institution of Hubei Cancer Hospital for scientific research paper. The studies were conducted in accordance with the local legislation and institutional requirements. The participants provided their written informed consent to participate in this study. Written informed consent was obtained from the individual(s) for the publication of any potentially identifiable images or data included in this article.

## Author contributions

LZ: Conceptualization, Project administration, Supervision, Writing – review & editing. YZ: Data curation, Formal analysis, Investigation, Software, Writing – review & editing. GC: Methodology, Resources, Writing – review & editing. FZ: Validation, Visualization, Writing – original draft. SL: Conceptualization, Supervision, Writing – original draft.
